# Associations of grandparental diabetes mellitus with grandchild BMI status

**DOI:** 10.1186/s12889-019-6485-y

**Published:** 2019-02-11

**Authors:** Yaping Lai, Juan Qi, Xingyong Tao, Kun Huang, Shuangqin Yan, Maolin Chen, Jiahu Hao, Fangbiao Tao

**Affiliations:** 1 Department of Maternal, Child and Adolescent Health, School of Public Health, Anhui Medical University, No. 81 Meishan Road, Hefei, 230032 Anhui China; 2 Anhui Provincial Key Laboratory of Population Health & Aristogenics, Hefei, China; 3 Teaching Center of Preventive Medicine, School of Public Health, Anhui Medical University, Hefei, China; 4 Ma’anshan Maternal and Child Health (MCH) Center, Ma’anshan, China

**Keywords:** Body mass index, Obesity, Diabetes, Infancy peak, Birth cohort

## Abstract

**Background:**

Maternal family history of diabetes was significantly and positively associated with birth weight in grandchildren, we aim to assess the effect of grandparental diabetes on the grandchild’ body mass index (BMI) at infancy peak (IP) and obesity status at age 2.

**Methods:**

In our study, family diabetes mellitus (DM) information from Ma’anshan Birth Cohort Study (MABC) were gathered. For children, height and weight were retrieved from medical records. BMI at 6 observations (0, 3, 6, 9, 12, 18 months) was plotted for every child. Onset of IP was determined by visual inspection. BMI at age 2 was categorized according to WHO Child Growth Standards as normal, overweight or obesity. The association between maternal grandfather’ diabetes and the grandchild’ BMI at IP and BMI at age 2 were tested using linear regression models and logistic regression models, respectively.

**Results:**

In our sample, about 6% of the maternal grandfather had DM, mean of infancy BMI peak was 18.37 kg/m^2^, and 6.6% of the children were obesity at age 2. Maternal grandfather with DM could significantly increase the IP BMI values (*β* = 0.30, 95 *CI* = 0.02~0.57), and was associated with obesity status at age 2 (OR = 1.92, 95 *CI* = 1.08~3.39), but maternal grandmother and paternal grandparents were unrelated.

**Conclusion:**

These results suggest that DM in maternal grandfather may be a risk factor for the grandchild high BMI at peak and obesity at age 2.

## Background

Unfavorable body mass index (BMI) at the early stage of human development can induce permanent health risks. The prevalence of childhood obesity has brought a heavy cost burden, result in later complications including diabetes, metabolic syndrome, and cardiovascular diseases [1–3]. One of the most critical periods proposed to be infancy BMI peak (IP), IP refers to the decrease in BMI following the maximum BMI at approximately 9 month of age [4], thus high BMI at IP could be used as a potential surrogate indicator for early development of obesity, and are predictive of later obesity [5, 6]. Despite its importance, little is known about trans-generational determinants of the BMI value at IP, especially regarding the family history of diabetes mellitus (DM).

Several studies have paid close attention to the intergenerational cycle of diabetes mellitus and obesity [7]. After all, there were estimated to be at least 451 million adults with diabetes worldwide in 2017. The number was expected to be approximately 693 million by 2045 [8]. Meanwhile, China now has the largest number of people with diabetes in the world and the number will keep growing [9]. Although, trans-generational inheritance of metabolic disease remains controversial, it is noticed that family history of diabetes was non-modifiable risk factor for the prevalence of obesity, metabolic syndrome and hypertension [10]. Moreover, diabetes in grandparents was a risk factor of diabetes in children [11], and studies have shown a clear association between grandparental DM and birth weight in grandchildren [12, 13]. Thereby, indicating that non-diabetic ancestors might be targeted in the prevention of early childhood obesity. However, previous studies investigated the effects of parental factors on the obesity in offspring [14], not much has been published about the grandparental DM.

There was evidence suggesting that maternal family history of diabetes were significantly and positively associated with gestational diabetes mellitus (GDM) [15], and GDM was associated with the risk of childhood obesity [16]. In view of the facts, it is interesting to determine whether grandparental DM is risk factor for high BMI peak and early childhood obesity.

## Methods

### Study population

**Fig. 1:**
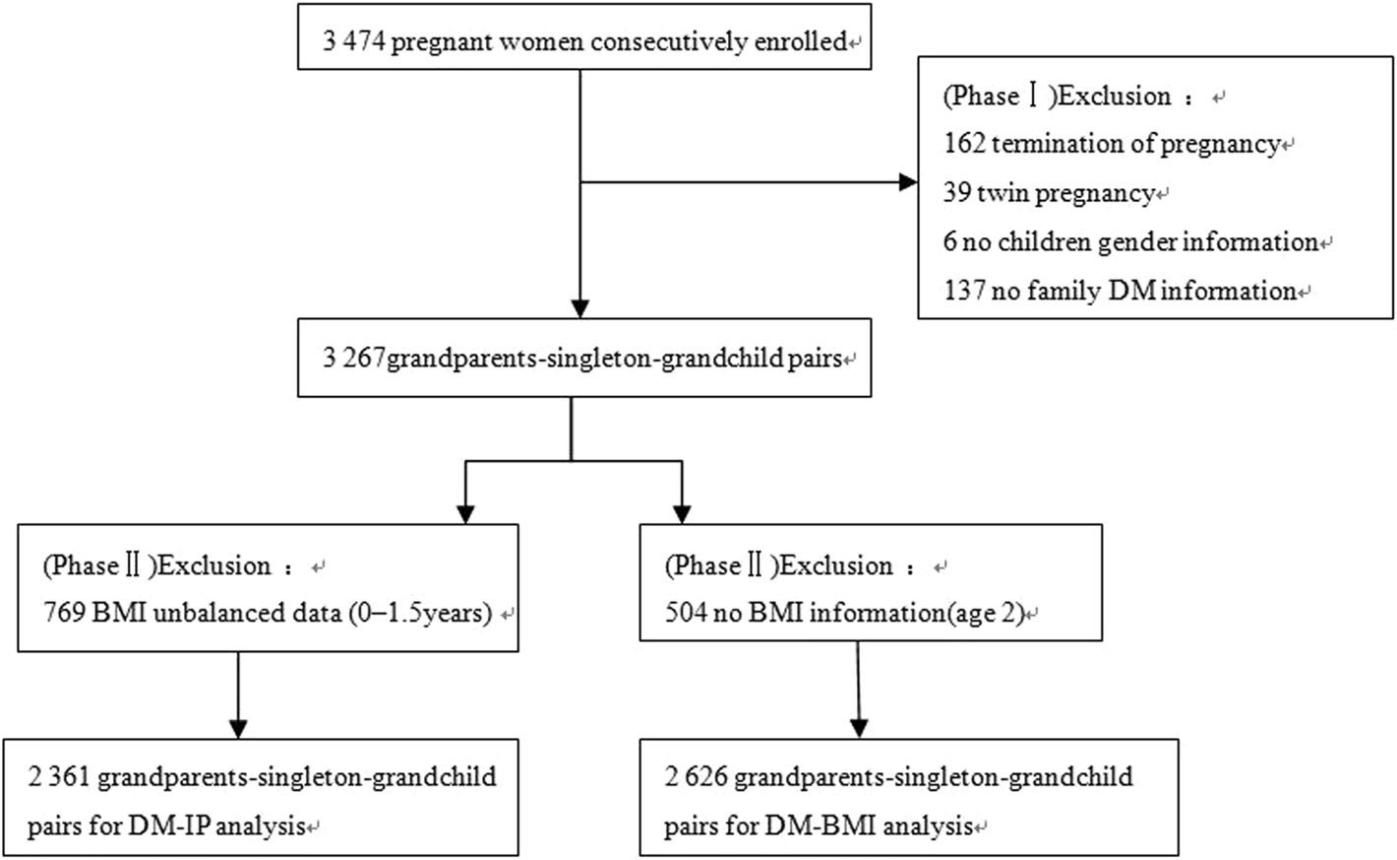
Inclusion and exclusion criteria of the participant flow

The Ma’anshan Birth Cohort Study (MABC) initially focused on the relationship between maternal factors during pregnancy and the development of the child. We looked into more information about lifestyle characteristic of father and grandparents. In this study, 3474 gravidas consecutively enrolled from May 2013 to September 2014 in Ma’anshan Maternal and Child Health(MCH) clinics when they did first physical examination during pregnancy. Details on the design and recruitment procedures have been described elsewhere [17]. Among the 3474 pregnant women who were invited, participants who presented with absence of live birth (*n* = 162), plural gestations (*n* = 39), without children gender information (*n* = 6), without grandparents exposure information (*n* = 137), unbalanced BMI data at 0–1.5 years of age (*n* = 769) or missed children’s BMI data at age 2 (*n* = 504) were excluded from this study (shown in Fig. 1), 2361 or 2626 grandparents-singleton-grandchild pairs were enrolled for the final data analysis. Ethical approval was provided by the Ethics Committee of the Anhui Medical University. Oral and written consents were collected from all participants.

#### Data collection

The information of ancestors and children was gathered by “Maternal and child health questionnaires”, which covered family medical history, sociodemographic characteristics, childhood lifestyles etc. DM in grandparents was based on self-reported data from questionnaires in which the mother was asked whether any of her biological parents and/or her parents in law had diabetes, and response categories for diabetes history were ‘yes’, ‘no’ and ‘unclear’. Children aged 0 months, 3 months, 6 months, 9 months, 12 months, 18 months and 24 months were measured by physician, therefore, their height and weight was extracted from medical records. BMI was calculated via dividing weight in kilograms by the square of height in meters.

**Fig. 2:**
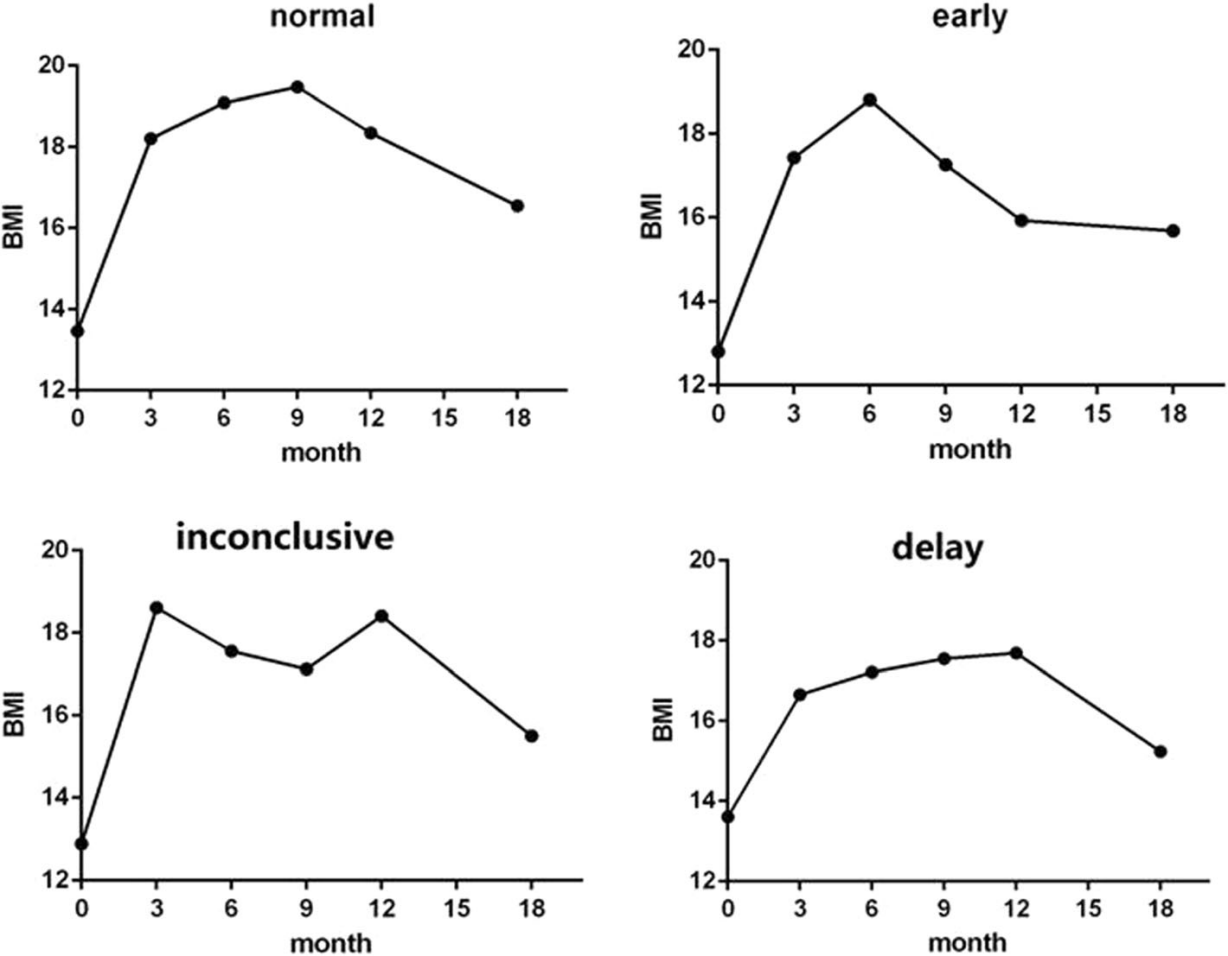
Four representative BMI growth trajectories

Individual -specific BMI peaks were determined by visual inspection. It has been proven that visual inspection appears to be preferable than polynomial models when estimating the milestone of BMI trajectories [18]. Onset of IP was defined as the maximum between the 0–1.5 years of age. We specified that each child should have all the six BMI observations and that at least 3 observations had to occur before or after the maximum to accurately establish the child specific trend. BMI data of infancy peaks were used as continues variables. If the peak occurred before 8 month or not earlier than 12 month, the child was said to have an early or delay IP [5]. Some children had BMI data that could not be categorized, and those children not to be included in the final analysis. Four representative growth trajectories were represented in Fig. 2. BMI at age 2 was categorized according to WHO Child Growth Standards (BMI-for-age for Birth to 2 years) and divided into three groups: normal weight was defined as BMI <85th percentile; overweight was defined as BMI < 95th percentile but ≥85th and obesity as ≥95th percentile.

### Co-variables considered

Based on other evidence, the following variables that might affect the physical development of children were considered: infant gender (boy or girl), feeding pattern(complementary feeding, bottle feeding or breastfeeding), monthly income(U.S. dollar), maternal education(high school and below, college and above), gestational diabetes mellitus (normal or GDM), hypertension disorders complicating pregnancy (HDCP) (normal or HDCP), maternal age at birth(< 25, 25–29 or ≥ 30 years old), maternal pre-pregnancy BMI(continuous variable), paternal pre-pregnancy BMI(continuous variable), gestational weight gain(continuous variable), production times(first or more than once), delivery mode(vaginal delivery or uterine-incision delivery), birth term (pre-term or born in time), birth weight(macrosomia, low birth weight or normal). Although analysing the data (*n* = 2361or 2626) without adjusting for feeding pattern, some maternal health parameters or some socio-economic covariates gave similar results, all these variables were adjusted for more accurate and more reliable.

### Statistical analysis and software

Descriptive statistics were used to examine the relationship between ancestral factors and early childhood BMI status. *T* test and analysis of variance (ANOVA) were applied to estimate the association between variables and BMI values at IP; We examined differences in subject characteristics amongst BMI classes by using *χ*^2^ tests. We used linear regression models to access the association between ancestors’ DM with grandchildren’ BMI at IP, and the association between maternal grandparents with DM and the grandchild’ BMI classes at age 2 were tested using multinominal logistic regression models. The associations were defined significant if *P* < 0.1. All analyses were conducted in SPSS 23.0.

## Results

Age and BMI at IP were not identifiable in 4.9% of our sample, mean of infancy BMI peak was 18.37 kg/m^2^(SD = 1.56) and occurred at 7.86 months. And about 6.6% of the children were obesity at age 2. The variables and BMI at IP or obesity status at age 2 of the 2361or 2626 grandparents-grandchild pairs are presented in Table 1. In our sample, about 6% of maternal or paternal grandfather and 4% of maternal or paternal grandmother had diabetes mellitus. It is worth pointing out that maternal grandfathers’ DM was associated with GDM (*P* = 0.006, *OR* = 1.80, 95% *CI*: 1.18 ~ 2.74). The mean values of BMI at IP by maternal grandfather category were 18.36 kg/m^2^ and 18.66 kg/m^2^; and the percentages of children with obesity by maternal grandfather category were 4.3 and 8.7% for maternal grandfathers with normal status and diabetes mellitus, respectively. The difference between both groups was statistically significant (*P* = 0.029, 0.080 respectively).

**Table 1:** Characteristics of BMI at IP and BMI class at age 2 by mean or percent

Characteristics	BMI at IP					BMI class at age 2				
	*N* (%)	Mean	SD	*P*	*N* (%)	Normal	Overweight	Obesity	*χ* ^*2*^	*P*
Maternal grandfather				0.029					5.04	0.080
normal	1962 (93.7)	18.36	1.56		2303 (93.9)	1867 (81.1)	291 (12.6)	145 (6.3)		
DM	131 (6.3)	18.66	1.58		149 (6.1)	112 (75.2)	21 (14.1)	16 (10.7)		
Maternal grandmother				0.205					3.12	0.211
normal	2068 (96.1)	18.37	1.57		2416 (96.1)	1954 (80.9)	304 (12.6)	158 (6.5)		
DM	85 (3.9)	18.58	1.33		97 (3.9)	74 (76.3)	18 (18.6)	5 (5.2)		
Paternal grandfather				0.499					0.53	0.768
normal	1693 (93.7)	18.36	1.55		1978 (93.6)	1585 (80.1)	263 (13.3)	130 (6.6)		
DM	113 (6.3)	18.47	1.71		135 (6.4)	111 (82.2)	15 (11.1)	9 (6.7)		
Paternal grandmother				0.888					0.38	0.827
normal	1826 (95.6)	18.36	1.55		2139 (95.7)	1722 (80.5)	283 (13.2)	134 (6.3)		
DM	85 (4.4)	18.39	1.42		96 (4.3)	78 (81.3)	11 (11.5)	7 (7.3)		
Infant gender				< 0.01					5.08	0.079
boy	1147 (51.0)	18.57	1.53		1350 (51.4)	1077 (79.8)	193 (14.3)	80 (5.9)		
girl	1101 (49.0)	18.16	1.56		1276 (48.6)	1036 (81.2)	149 (11.7)	91 (7.1)		
Birth term				0.174					1.64	0.441
preterm	80 (3.6)	18.61	1.50		107 (4.1)	81 (75.7)	17 (15.9)	9 (8.4)		
in time	2168 (96.4)	18.36	1.56		2519 (95.9)	2032 (80.7)	325 (12.9)	162 (6.4)		
Maternal education				0.732					0.08	0.962
high school and below	877 (39.0)	18.36	1.54		1056 (40.2)	847 (80.2)	139 (13.2)	70 (6.6)		
college and above	1371 (61.0)	18.37	1.57		1570 (59.8)	1266 (80.6)	203 (12.9)	101 (6.4)		
GDM				0.477					11.72	< 0.01
no	1963 (87.3)	18.36	1.55		2285 (87.0)	1862 (81.5)	282 (12.3)	141 (6.2)		
yes	285 (12.7)	18.43	1.63		341 (13.0)	251 (73.6)	60 (17.6)	30 (8.8)		
HDCP				0.075					4.72	0.095
no	2110 (93.9)	18.36	1.55		2457 (93.6)	1986 (80.8)	311 (12.7)	160 (6.5)		
yes	138 (6.1)	18.60	1.63		168 (6.4)	126 (75)	31 (18.5)	11 (6.5)		
Production times				0.047					4.79	0.091
first	2040 (90.7)	18.35	1.57		2388 (90.9)	1934 (81.0)	304 (12.7)	150 (6.3)		
others	208 (9.3)	18.58	1.44		238 (9.1)	179 (75.2)	38 (16.0)	21 (8.8)		
Delivery mode				0.004					10.22	< 0.01
vaginal delivery	1114 (49.6)	18.28	1.47		1297 (49.4)	1076 (83.0)	147 (11.3)	74 (5.7)		
caesarean section	1134 (50.4)	18.47	1.63		1328 (50.6)	1036 (78.0)	195 (14.7)	97 (7.3)		
Feeding pattern				0.052					9.07	0.059
Complementary feeding	625 (28.3)	18.35	1.47		739 (28.7)	602 (81.5)	95 (12.9)	42 (5.7)		
Bottle feeding	510 (23.1)	18.24	1.43		581 (22.6)	448 (77.1)	95 (16.4)	38 (6.5)		
breastfeeding	1076 (48.7)	18.44	1.66		1254 (48.7)	1020 (81.3)	146 (11.6)	88 (7.0)		
Monthly income				0.321					11.83	0.019
< 360.5	594 (26.4)	18.40	1.54		705 (26.8)	553 (78.4)	114 (16.2)	38 (5.4)		
360.5–576.8	939 (41.7)	18.41	1.57		1107 (42.2)	889 (80.3)	134 (12.1)	84 (7.6)		
> 576.8	715 (31.8)	18.30	1.56		814 (31.0)	671 (82.4)	94 (11.5)	49 (6.0)		
Maternal age				0.965					1.96	0.743
18–24	632 (28.1)	18.39	1.57		748 (28.5)	606 (81.0)	97 (13.0)	45 (6.0)		
25–29	1205 (53.6)	18.37	1.57		1403 (53.4)	1122 (80.0)	190 (13.5)	91 (6.5)		
≥30	411 (18.3)	18.37	1.51		475 (18.1)	385 (81.1)	55 (11.6)	35 (7.4)		
Birth weight				< 0.01					23.29	< 0.01
normal	2032	18.31	1.53		2355 (89.7)	1920 (81.5)	291 (12.4)	144 (6.1)		
low birth weight	40	18.14	1.24		58 (2.2)	48 (82.8)	6 (10.3)	6 (6.9)		
macrosomia	176	19.16	1.77		212 (8.1)	144 (67.9)	45 (21.2)	23 (10.8)		
Residence				0.565					4.77	0.57
city proper	1808 (80.4)	18.37	1.57		2096 (79.8)	1691 (80.7)	274 (13.1)	131 (6.3)		
suburbs	239 (10.6)	18.48	1.52		283 (10.8)	230 (81.3)	32 (11.3)	21 (7.4)		
county town	40 (1.8)	18.21	1.96		37 (1.4)	29 (78.4)	7 (18.9)	1 (2.7)		
countryside	161 (7.2)	18.29	1.54		210 (8.0)	163 (77.6)	29 (13.8)	18 (8.6)		
Paternal BMI		23.44	3.56							
Maternal BMI		20.93	2.85							
GWG		17.93	5.01							

*BMI* body mass index, *IP* infancy peak, *GDM* gestational diabetes mellitus, *GWG* gestational weight gain, *HDCP* hypertensive disorder complication pregnancy

The association of grandparents’ diabetes mellitus and children’ BMI at IP are shown in Table 2. Maternal grandfather with diabetes mellitus was directly related to higher BMI values (*β* = 0.31, 95% *CI* = 0.03~0.58), a similar trend when the estimating was performed using a multivariate analysis (*β* = 0.30, 95% *CI* = 0.02~0.57); the results suggest that BMI increases as when maternal grandmother and paternal grandparents had diabetes mellitus, however, the analysis did not reach a statistically significant difference.

**Table 2:** Regression coefficient of BMI at IP depending on ancestors’ diabetes mellitus

Ancestors exposed	Univariate analyses		Multivariate analyses ^a^	
	*β*	95% *CI*	*β*	95% *CI*
Maternal grandmother	0.22	−0.12~0.56	0.16	−0.17~0.50
Maternal grandfather*	0.31	0.03~0.58	0.30	0.02~0.57
Paternal grandmother	0.02	−0.31~0.36	− 0.07	− 0.40~0.27
Paternal grandfather	0.10	−0.20~0.40	0.10	−0.19~0.40

a: Adjusted for infant gender, birth term, feeding pattern, monthly income, education, GDM, HDCP, maternal age, maternal pre-pregnancy BMI, paternal BMI, GWG, production times, delivery way

*BMI* body mass index, *IP* infancy peak, *GDM* gestational diabetes mellitus, *GWG* gestational weight gain, *HDCP* hypertensive disorder complication pregnancy

*P<0.05

Children’ obesity and overweight were compared with normal weight across different ancestor exposure (see Table 3). In fully adjusted models, maternal grandfather’ diabetes mellitus predicted children obesity (*OR* = 1.92, 95% *CI* = 1.08~3.39), no statistical difference between maternal grandfather’ diabetes mellitus and children overweight (*OR* = 1.25, 95% *CI* = 0.76~2.06); however, maternal grandmother and paternal grandparents with diabetes mellitus was unrelated to children BMI status at age 2.

**Table 3:** Odd ratio of BMI at age 2 depending on ancestors’ diabetes mellitus

Ancestors exposed	Univariate analyses OR(95%CI)		Multivariate analyses OR(95%CI)^a^	
	obesity	overweight	obesity	overweight
Maternal grandmother	0.84 (0.33~2.10)	1.56 (0.92~2.65)	0.73 (0.28~1.87)	1.32 (0.76~2.31)
Maternal grandfather	1.84 (1.06~3.19)*	1.20 (0.74~1.95)	1.92 (1.08~3.39)*	1.25 (0.76~2.06)
Paternal grandmother	1.15 (0.52~2.55)	0.64 (0.86~1.63)	1.18 (0.52~2.66)	0.79 (0.40~1.57)
Paternal grandfather	0.99 (0.49~2.00)	0.81 (0.47~1.42)	1.03 (0.50~2.10)	0.78 (0.44~1.40)

a: Adjusted for infant gender, birth term, feeding pattern, monthly income, education, GDM, HDCP, maternal age, maternal pre-pregnancy BMI, paternal BMI, GWG, production times, delivery way

*BMI* body mass index, *IP* infancy peak, *GDM* gestational diabetes mellitus, *GWG* gestational weight gain, *HDCP* hypertensive disorder complication pregnancy

*P<0.05

## Discussion

Our results demonstrate that BMI at IP and obesity status at age 2 were under the influences of DM in maternal grandfather, and the influences were independent of maternal age, parental BMI, maternal education, gender, birth-term, family monthly income, et al. However, we did not find an association between paternal grandparental DM or maternal grandmother DM and children’ BMI status either at IP or at age 2.

Currently, there is a lack of putative criteria to define excess or normal BMI value at IP. In an early study [19] involving healthy term infants in shanghai, China, Zhuochun Wu et al. investigated that the mean magnitude of BMI peak was 18.33 kg/m^2^, which comparable to our study. To our knowledge, our study is among the first to provide evidence that family history of DM, particularly DM in maternal grandfather, can potentially alter the BMI values at IP as well as BMI status at age 2 of grandchildren. Our data further substantiate the feasibility of trans-generational inheritance of metabolic disease.

Previous studies have only examined associations of GDM or other maternal factors with offspring BMI status and infancy BMI peak, but not grandparental DM. In concordance with our study, a cross sectional national survey in Sri Lanka (*n* = 4485) demonstrated that family history of diabetes was associated with the prevalence of adults obesity as defined with the use of BMI [10]. Likewise, the Avon longitudinal study of parents and children (ALSPAC) (*n* = 12,076) revealed that the grandchildren of maternal grandparents with DM were more likely to have higher birth weight than those grandchildren of non-grandparental diabetics [12], and a recent study in Netherlands indicated that children with strong family history of diabetes had higher total cholesterol [20]. In contrast, no association was observed between metabolic syndrome including obesity, insulin resistance, and glucose intolerance in F0 mice and obesity in grand-offspring (F2) by using a mouse model [21]. However, the animal study focus on the metabolic analysis of males only, namely, paternally-induced trans-generational effects, but not maternally-induced trans-generational effects. Taken together, results from the aforementioned studies and from our study showed that maternal grandparental DM may have a greater influence on grand-offspring BMI status than does paternal grandparental DM.

The biological bases underlying the grandparental diabetes with their grandchildren growth are not known. We assume that genomic imprinting could passed from maternal grandfathers to mothers and then to their grandchildren, for maternal grandfathers’ DM was associated with GDM, and GDM were risk factor for offspring’ obesity. Recently, studies have taking DNA methylation as the primary mechanism of trans-generational of diverse metabolic disease [22, 23]. However, owing to the experimental limitations or other reasons, most studies have only focused on epigenomic variants by measuring the epigenome in somatic tissue or in germ cells [24], which presents a very limited picture of the potential mechanisms. Moreover, DNA methylation is definitely not the only inherited mark correlated with metabolic disease [25], there are a lot of questions to answer when keenly access the phenomenon of epigenetic inheritance [26]. Also, this cross-generation associational of diabetes and obesity may relate to shared cultural socio-economic environments, could be suffering from malnutrition.

This study has several limitations. First and foremost, the family diabetes history was self-reported by the mothers, therefore we have no idea what kind of diabetes (type 1 or type 2) the grandparents were affected with. What’s worse, it is regrettable that our study has no concern with the population with undiagnosed diabetes or prediabetes. After all, a significant proportion remaining undiagnosed (over 60% were unaware of their diagnosis) in China [27], partly because the poor public health education. However, the prevalence of grandparental diabetes in our study was consistent with the findings of The China Chronic Disease and Risk Factors Surveillance study, which presented that weighted diagnosed diabetes (defined as a self-reported diagnosis) prevalence of the 40–59 age group Chinese population was about 5.0% [28]. Secondly, we didn’t consider the children’s physical activity data or dietary data, and this could have affected the estimates. Thirdly, because of missing data on children’s BMI information, one third of the participants were excluded from the final analysis. Although the characteristics between the included and the total participants were similar (Table 4), the exclusion could bias the results. Next, this study was based on data collected from a municipal health hospital for women and children, the recruited sample were women who were willing to undergo their prenatal care and delivery in the centre, most of them live in city proper (approximately 80%), the sampling design might be a limitation. Finally, we merely used BMI as proxies for physical growth, other indicators that represent growth and development, such as skinfold thicknesses, should consider to be implemented.

**Table 4:** Characteristics between the included and the total participants

Characteristics	*N*(%) or mean(SD)		*χ* ^*2*^ */F*	*P*
	Total participants	2361 sample		
Monthly income			0.379	0.827
< 360.5	866(26.5)	623(26.4)		
360.5–576.8	1400(42.9)	997(42.2)		
> 576.8	1001(30.6)	741(31.4)		
Residence			4.520	0.210
city proper	2548(78.0)	1893(80.2)		
suburbs	386(11.8)	255(10.8)		
county town	56(1.7)	41(1.7)		
countryside	277(8.5)	172(7.3)		
Infant gender			0.048	0.826
boy	1666(51.0)	1211(51.3)		
girl	1601(49.0)	1150(48.7)		
Birth term			0.769	0.381
preterm	134(4.1)	86(3.6)		
in time	3133(95.9)	2275(96.4)		
Maternal grandfather age	54.35(5.887)	54.48(5.891)	0.000	0.999
	Total participants	2626 sample		
Monthly income			0.290	0.865
< 360.5	866(26.5)	705(26.8)		
360.5–576.8	1400(42.9)	1107(42.2)		
> 576.8	1001(30.6)	814 (31.0)		
Residence			3.266	0.352
city proper	2548(78.0)	2096(79.8)		
suburbs	386(11.8)	283(10.8)		
county town	56(1.7)	37(1.4)		
countryside	277(8.5)	210(8.0)		
Infant gender			0.100	0.752
boy	1666(51.0)	1350(51.4)		
girl	1601(49.0)	1276(48.6)		
Birth term			0.003	0.959
preterm	134(4.1)	107(4.1)		
in time	3133(95.9)	2519(95.9)		
Maternal grandfather age	54.35(5.887)	54.45(5.905)	0.002	0.967

## Conclusions

In conclusion, we found that DM in maternal grandfather may be a risk factor for the grandchild high BMI at infancy peak and obesity at age 2, and these population epidemiological findings may to some extent draw attention to trans-generational effects of diabetes and obesity.

## Abbreviations

BMI: Body mass index
DM: Diabetes mellitus
GDM: Gestational diabetes mellitus
HDCP: Hypertension disorders complicating pregnancy
IP: Infancy BMI peak
MABC: Ma’anshan Birth Cohort Study
